# What factors are driving the rapid growth of education levels in China?

**DOI:** 10.1016/j.heliyon.2023.e16342

**Published:** 2023-05-15

**Authors:** Lidan Yang, Shixiong Cao

**Affiliations:** School of Economics, Minzu University of China, No. 27 Zhongguancun South Street, Haidian District, Beijing, 100081, China

**Keywords:** Education policies, Worker skills, Socioeconomic development, Geographic heterogeneity, Education level, China

## Abstract

Recognizing the critical elements that promote improvement of a country's education level (here, the mean number of years of education) is a necessary prerequisite for developing policies and plans to promote the long-term development of education and the people's quality of life. By identifying the factors that constrain the development of education and the strength of each factor's influence, we aimed to provide theoretical support and practical suggestions for advancing the development of education in China and other countries. We collected data related to China's education sector from 2000 to 2019, identified the key factors driving the per capita number of years of education of Chinese nationals, quantified their degree of influence on education, and investigated the association of each factor with the per capita education in different regions using sub-regional regression and geographic and time-weighted regression models. We found that per capita GDP, education funding, and urbanization promoted educational attainment, whereas allowing the student–teacher ratio to increase decreased educational attainment. Therefore, promoting the development of education requires that the government take measures to promote economic and social development, increase the financial investment in education, and train more high-quality teachers who can work in regions that currently lack sufficient teachers. In addition, the existence of regional heterogeneity means that both central and local governments must fully account for local realities when they formulate education policies and tailor them to local conditions.

## 1 Introduction

Education is a core element for improving lives and promoting socioeconomic development. As a result, it has been a focus of attention for governments around the world. The UN vision “Transforming Our World: The 2030 Agenda for Sustainable Development” (https://sdgs.un.org/2030agenda) includes education as one of the priorities to support sustainable development and explicitly states the need to “ensure inclusive and equitable quality education and promote lifelong learning opportunities for all” [1]. Education has a significant impact on a person's work opportunities and the quality of their life, so it is widely recognized as the key to opening the door to personal development and to overcoming poverty [2]. Through education, human and civic values are preserved and passed on to future generations [3], and the current situation of both societies and individuals can be improved by education [4]. Improving a nation's education level is not only crucial to the productivity and life of the present generation, but is also crucial to the vital interests of the next generation and the long-term development of society [5].

**Fig. 1:**
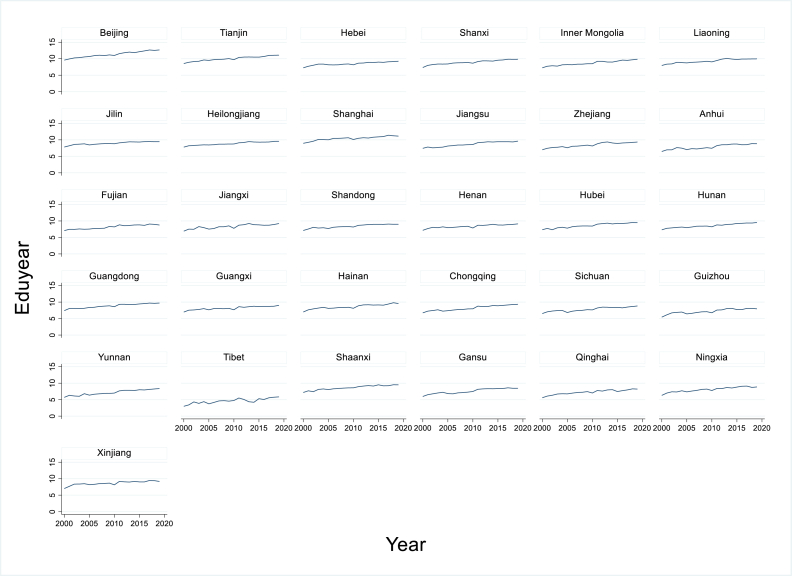
Changes in the per capita number of years of education (*Eduyear*) between 2000 and 2019 in 31 Chinese provinces.

In the past few decades, China's education system has developed rapidly, and the per capita education level has increased in parallel (Fig. 1). Since the establishment of the Chinese communist government in 1949, China has generally worked to eliminate illiteracy and promote basic education and, since the late 1970s, has aggressively promoted university education, and has obtained remarkable results. For example, the per capita number of years of education for Chinese adults of working age was only 1.6 in 1949, but this value reached 10.9 in 2021 [6]. China has not only achieved 9 years of compulsory education for most citizens [7], but has also seen significant growth of higher education [8]. However, the average number of years of education for the Chinese population aged 6 and above is still less than 9.5 years, which is far below the average of 12.7 years in developed countries [5]. Closing this gap is of great practical significance to improve the national education level, thereby improving both worker training and the quality of life.

Identifying the factors that constrain the development of education and the strength of their impact is essential to support efforts to improve the educational system, enhance the quality of education, and optimize educational policies. Scholars from the international community have conducted long-term studies and have demonstrated that economic growth [9,10], transportation accessibility [11], educational investment [[12], [13], [14]], the number and quality of teachers [15], and urbanization [16] strongly influenced the educational development of a society. However, many of the factors that affect educational attainment have been explored in isolation. For example, two recent studies explored the impact on education only in terms of a single dimension of economic [17] and educational [13] inputs. Because such studies focused on individual factors, they failed to provide a comprehensive analysis. In addition, most researchers used cross-sectional data or data from a short time series, and therefore failed to reflect the whole picture created by the many factors that affect educational development; furthermore, the pooled regression methods used by some scholars have potentially serious endogeneity problems [18,19]. Moreover, the results of previous studies failed to quantify the strength of each factor's influence on education. For example, two relatively recent studies did not analyze the relative importance of each factor that was included in their studies [12,20]. Although we have learned from the insights provided by these and other previous studies, we designed the present study to account for the abovementioned problems: we provide a more comprehensive perspective on the different factors that influence education development and the strength of their influence, using long-term regional (provincial) data to allow a comparison among regions that revealed both the dominant factors and their relative strengths. Our goal was to promote the development of education in China as a whole and improve the development in different regions of China with different characteristics and needs.

Awareness of the key factors that affect a person's education level is an essential prerequisite for promoting the transformation and upgrading of a country's economic, technological, and social spheres [21,22]. By cultivating intrinsic human qualities such as the desire to learn and improving a range of competencies (e.g., cognitive abilities, problem-solving skills, learning efficiency), education can significantly increase someone's quality of life and their ability to contribute to their nation's future [23]. Improvement of worker skills is, in turn, positively correlated with national technological progress [24]. Living in the current technological century, no country can succeed against fierce international competition without strong educational outcomes [25]. Given China's size, understanding the efforts made to promote the development of Chinese education will provide an important reference for other developing countries around the world that are seeking to improve their education policies and the quality of education.

## 2 Theoretical framework

To obtain a more scientific and comprehensive understanding of the key factors affecting educational attainment and to ensure the reliability and feasibility of the research results, we identified and analyzed the driving factors that affect the educational attainment of Chinese nationals in three steps. First, we identified factors that can potentially influence educational development. To do so, we screened indicators from the literature that researchers found could affect education along multiple dimensions. Based on this analysis, we selected four indicators (the student–teacher ratio, per capita education expenditure, per capita GDP, and urbanization level) as explanatory variables, and chose a fixed-effects model (which provided better results than pooled and random-effects regressions) to quantify their strengths. Second, we analyzed the relative importance of the different factors for influencing educational attainment by quantifying the strength of each factor using a contribution analysis model. Third, we performed our analysis separately for each province and for three regions of China, and used the results to characterize the regional heterogeneity of the key factors that influenced educational attainment using sub-regional tests and geographic and time-weighted regression (GTWR) models.

By examining the drivers of per capita educational attainment in 31 Chinese provinces, we identified the factors that may affect education in China as a whole and in different regions as well as the strengths of their effects. On this basis, we analyzed the possible causes of educational attainments and made targeted recommendations that will contribute to improving the Chinese population's educational attainment. The results of our study will not only help to improve the educational attainment of Chinese citizens, but will also serve as an important reference for the improving of education, worker skills, and the quality of life in other developing countries facing similar situations.

## 3 Methods

### 3.1 Study design and data sources

We analyzed the factors influencing the educational attainment of Chinese nationals and the relative importance of the different factors from both an overall perspective (for China as a whole) and from regional (western, central, and eastern China) and sub-regional (provincial) perspectives using panel fixed-effects models and GTWR models, respectively. We chose these paths to improve the precision and reliability of our results, and to make our findings more relevant for developing feasible measures for advancing the development of education at the national and local levels.

We obtained data on education and the driving factors for 31 Chinese provinces, including province-level municipalities (e.g., Beijing) and autonomous regions, from 2000 (the earliest year for which data was available for all of China) to 2019 to exclude the impact of the COVID-19 pandemic, which began to have a serious impact in 2020. (Hereafter, we will refer to them all as “provinces” for simplicity.) Our data selection was based on availability (since our goal was to compare 31 provinces with different data availability) and on objectivity (since it was necessary to use comparable data from each province). We excluded Hong Kong, Macao, and Taiwan from the analysis since the required data was not available for the whole study period and these regions have both more educational support and different educational policies. We obtained the data from the China Statistical Yearbooks from 2000 to 2019 for each province, which were provided by the National Bureau of Statistics of China from 2001 to 2020 (http://www.stats.gov.cn/). Our map data were derived from the Resources and Environment Data Center of the Chinese Academy of Sciences (http://www.resdc.cn).

### 3.2 Indicator selection

For the predicted variable that represents educational attainment, we chose the per capita number of years of schooling of the Chinese population aged 6 years and above. (Hereafter, we will refer to this as the “per capita years of education” for simplicity.) We calculated this using the following formula:

(1) $$y_{it}=E/P$$

where *y*_*it*_ represents the average years of schooling at age 6 and older in province *i* in year *t. E* is the sum of the population in a given school grade multiplied by the corresponding number of years of schooling for students who reached that grade, which is calculated as follows: the number of elementary school students × 6 years + the number of junior high school students × 9 years + the number of senior high school students × 12 years + the number of college students × 15 years + the number of undergraduate university students × 16 years + the number of graduate students × 19 years. *P* is the total number of people aged 6 years and over.

We chose nine explanatory variables based on the results of previous studies and China's national conditions, with variables that represented economic, social, and policy perspectives (Table 1): transportation accessibility [11], urbanization [16], marketization [8], the proportion of registered unemployed workers [10], industrial structure [26], birth rate [20], per capita education expenditure [12], the elementary school student–teacher ratio (hereafter, referred to as the student–teacher ratio for simplicity) [14], and per capita GDP [27]. To let us combine all variables in our analysis despite their different magnitudes and units of measurement, we first standardized the data using *z*-scores. Subsequently, we employed stepwise regression to separately screen the explanatory variables and tested the model goodness of fit (*R*^2^), statistical significance of the variables (the *p* level), and statistical significance of the equations to eliminate nonsignificant variables and avoid multicollinearity among the explanatory variables that would reduce the robustness of the regression results. The criteria for retaining a variable were a variance inflation factor (*VIF*) < 7.5, statistical significance at *p* < 0.05, and passing of a robustness test for lagged one-period independent variables as instrumental variables. After screening, we retained only four explanatory variables: the student–teacher ratio, per capita education expenditure, per capita GDP, and urbanization level.

**Table 1:** Indicator selection and explanations of their meaning. All data were obtained from the National Bureau of Statistics of China from 2001 to 2020 (http://www.stats.gov.cn/).

Indicators	Implication	Description
*Eduyear*	Per capita years of education	Average years of schooling at age 6 and older
*PGDP*	Per capita GDP	Regional GDP/total population of the region (yuan/person)
*STR*	Student–teacher ratio	Number of elementary school students divided by the number of full-time teachers
Expenditure	Per capita education expenditure	Per capita education spending in the region (based on the sum of education spending from all sectors rather than just government funding for education)
Urbanization	Regional urbanization level	Ratio of the urban population to the total population
Unemployment	Registered unemployment rate	Number of registered unemployed workers in cities/total population old enough for employment
Marketization	Degree of marketization	1– (number of employees in urban state-owned enterprises and collective-owned units/total number of urban employed persons)
Transportation	Transportation accessibility	Length of highways/regional area (km/km^2^)
Birth rate	Population birth rate	The number of births in a given year/the total population of the province in that year
Industry	Industrial structure	Value added by tertiary industries/provincial GDP

### 3.3 Methodology

Using the indicators identified in section 3.2, we performed regression analysis using version 16 of the Stata software (https://www.stata.com/), and implemented the Hausman test for model selection using a pooled regression model, a fixed-effects model, and a random-effects model (Table 2). The results indicated that the fixed-effects model was the most appropriate method for our study:

(2) $$y_{it}=\alpha_{i}+\sum_{k=1}^{n}\beta_{k}x_{kit}+z_{i}+\varepsilon_{it}$$

where *y*_*it*_ represents the per capita years of education in province *i* in year *t*, which is calculated from equation (1), α is the *y*-intercept, β_*k*_ is the regression coefficient for indicator *k*, *x*_*kit*_ is the value of independent variable *k* that affects the years of education in province *i* for year *t*, *z*_*i*_ is the comprehensive influence on *y* of all non-time variables in province *i* that have not been observed, and ε_*it*_ is a random error term for province *i* and year *t*.

**Table 2:** Screening of the models. *PGDP*, per capita GDP; *STR*, student–teacher ratio.

Variables	Pooled regression	Fixed effects	Random effects
*STR*	−0.245***	−0.180***	−0.177***
	(0.035)	(0.023)	(0.023)
Expenditure	−0.342***	0.173***	0.147***
	(0.052)	(0.032)	(0.032)
*PGDP*	0.546***	0.323***	0.325***
	(0.067)	(0.035)	(0.035)
Urbanization	0.720***	0.332***	0.384***
	(0.051)	(0.049)	(0.048)
Constant	8.422***	8.422***	8.422***
	(0.026)	(0.011)	(0.102)
Hausman test statistic		25.82	
*p*-value		0.0001	

Notes: Standard errors are presented in parentheses. *, **, and *** represent significance at *p* < 0.10, 0.05, and 0.01, respectively.

To ensure the robustness of the study results, we corrected the standard errors using White's heteroskedasticity (i.e., heteroskedasticity-robust standard errors) to deal with heteroskedasticity and serial correlation. In addition, we conducted a contribution analysis to quantify the strength of each indicator using the absolute values of the regression coefficients:

(3) $$Con_{k}=\frac{AC_{k}}{\sum_{k=1}^{n}AC_{k}}\times 100％$$

where *Con*_*k*_ represents the contribution of explanatory variable *k* to the education level, and *AC*_*k*_ represents the absolute value of the regression coefficient (β_*k*_) from equation (2).

**Fig. 2:**
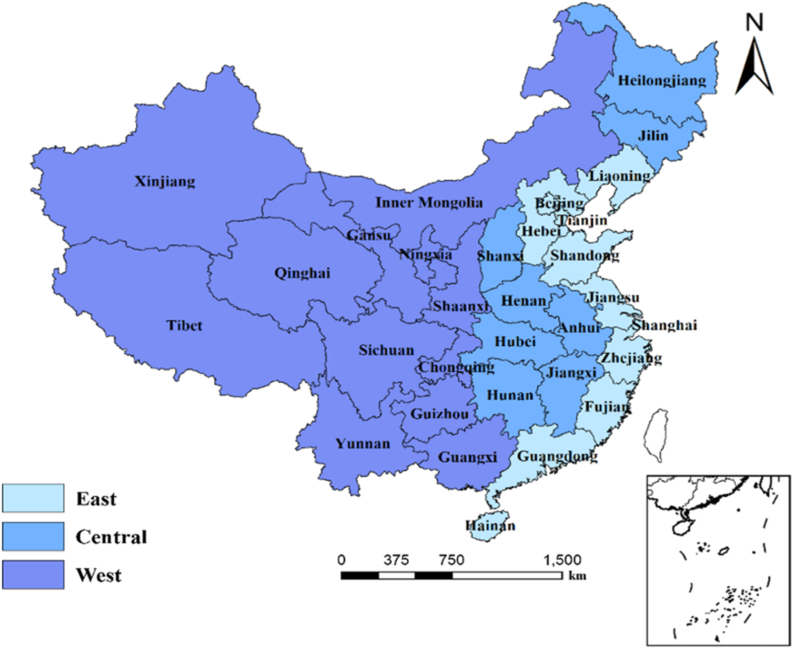
The provinces included in eastern, central, and western China. Regions were defined by China's National Bureau of Statistics based on the economic level and geographic location.

To analyze the regional differences in the factors influencing the per capita years of education, we divided the selected data into three regions (eastern, central, and western China) according to the criteria of the National Bureau of Statistics of China (Fig. 2), and analyzed the influence of the four indicators on the per capita years of education in each region and province. In addition, to account for the spatial and temporal differences in the strength of the indicators, we used a recent GTWR model [28] to explore the relationship between per capita educational attainment and its influencing factors, and then used version 10.8 of the ArcGIS software (www.esri.com) to construct and map the GTWR model. The model has the following general structure:

(4) $$y_{i}=\beta_{0}\left(\mu_{i},v_{i},t_{i}\right)+\sum_{k=1}^{n}\beta_{i}\left(\mu_{i},v_{i},t_{i}\right)x_{ik}+\varepsilon_{i}$$

where *y*_*i*_ is the dependent variable (per capita years of education) in province *i*; *u*_*i*_, *v*_*i*_, and *t*_*i*_ are the longitude, latitude, and year in province *i*, respectively (i.e., the spatial and temporal positions of the data); **β**_0_ (μ_*i*_, *v*_*i*_, *t*_i_) is the intercept value; *x*_*ik*_ is the value of indicator *k* in province *i*; **β**_*k*_ (μ_*i*_, *v*_*i*_, *t*_*i*_) is the regression coefficient for indicator *k* in province *i*; and ε_*i*_ is a random error term for province *i*.

## 4 Results

**Fig. 3:**
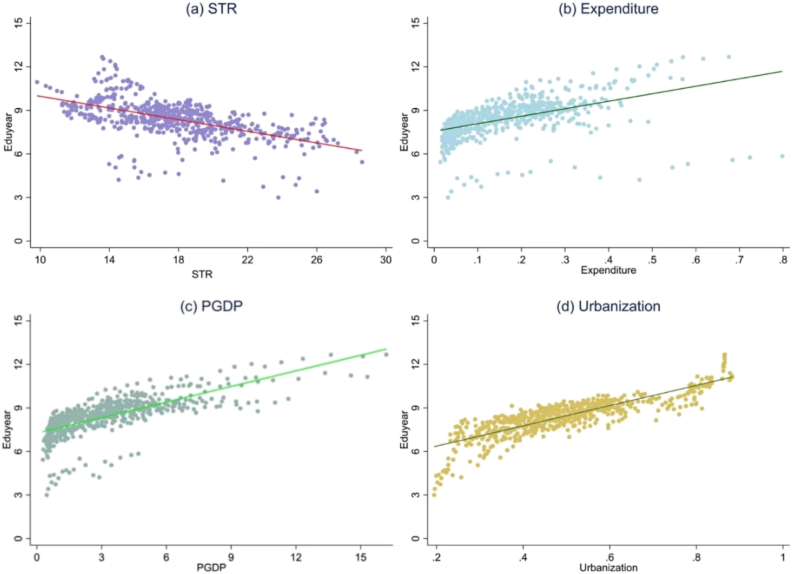
Regression results for the relationship between each explanatory variable and the response variable (per capita years of education, *Eduyear*) based on data from 2000 to 2019. Explanatory variables: (a) *STR*, the student–teacher ratio; (b) expenditure on education; (c) *PGDP*, per capita GDP; (d) urbanization (% of population living in an urban area).

The regression analysis for the relationship between each explanatory variable and per capita years of education (Fig. 3a–d) showed that increasing the student–teacher ratio (*STR*) significantly decreased the years of education, whereas increasing the expenditures, the per capita GDP (*PGDP*), and urbanization significantly increased years of education.

From a national perspective, with all provinces combined (Table 3), *PGDP* (the core indicator of regional economic development) and urbanization had the greatest impact on education, with contributions of 32.0 and 32.9%, respectively, for the whole study period; that is, they together accounted for roughly two-thirds of the total effect. *PGDP* represents the government's ability to provide financial support such as payment of tuition fees for students, and can therefore increase the per capita years of education. Urbanization provides a convenient environment for the development of education and reduces the transportation and time costs for students. The expenditure on education provides similar support, as well as increasing infrastructure investment (e.g., classrooms); this explains its contribution of 17.2%. In contrast, *STR* had a strong negative relationship with years of education; this is logical becausewhen there are too many students per teacher, the teacher cannot spend enough time with each student to meet their needs or increase their enthusiasm for learning. When we divided the regression into two time periods to account for changes in influencing factors before and after the full implementation of universal education in 2011, the results were broadly similar, but with different contributions. In both periods, *PGDP* remained the most important factor. However, expenditure became much more important than urbanization from 2000 to 2010, and the two factors were equally important from 2010 to 2019. In addition, after the full popularization of a compulsory 9-year education, the contradiction between the student–teacher ratio and education attainment eased, and *STR* was no longer a statistically significant factor that affected the level of education.

**Table 3:** Regression results for the drivers of per capita years of education at the national level for the study period as a whole and for two time periods. *STR*, student–teacher ratio; *PGDP*, per capita GDP.

	2000–2019		2000–2010		2011–2019	
	Regression coefficient	Contribution (%)	Regression coefficient	Contribution (%)	Regression coefficient	Contribution (%)
*STR*	−0.180***	17.86	−0.122**	11.49	0.030	6.19
	(0.03)		(0.05)		(0.06)	
Expenditure	0.173**	17.16	0.350**	32.96	0.149***	30.72
	(0.06)		(0.15)		(0.03)	
*PGDP*	0.323***	32.04	0.432***	40.67	0.156**	32.16
	(0.08)		(0.12)		(0.06)	
Urbanization	0.332***	32.94	0.158	14.88	0.150*	30.93
	(0.09)		(0.10)		(0.09)	

Notes: Standard errors are presented in parentheses. The regression coefficient (βk) was calculated using equation (2), and the contribution was calculated using equation (3), as detailed in the Methods section. *, **, and *** represent significance levels of *p* < 0.10, 0.05, and 0.01, respectively.

Because it takes time for the effects of some factors to be seen, we analyzed the effect of time lags ranging from 1 to 3 years on the regression results (Table 4). The effects were broadly similar to those in Table 1 (without a time lag): *PGDP* and urbanization remained the most important factors that promoted years of education and together, they accounted for more than half of the total effect with all three time lags. The contributions of economic growth (*PGDP*) and education expenditure increased with an increasing time lag, reaching their maximum values in the third year (36.3 and 20.2%, respectively); in contrast, the contribution of urbanization decreased over time, but remained the second-most-important factor. The contribution of *STR* fluctuated, but remained negative for all time lags.

**Table 4:** Regression results after accounting for a time-lag effect, using data for the whole study period from 2000 to 2019. *STR*, student–teacher ratio; *PGDP*, per capita GDP.

	Lag of 1 year		Lag of 2 years		Lag of 3 years	
	Regression coefficient	Contribution (%)	Regression coefficient	Contribution (%)	Regression coefficient	Contribution (%)
*STR*	−0.146***	15.37	−0.139***	14.70	−0.143***	15.20
	(0.03)		(0.03)		(0.04)	
Expenditure	0.172***	18.10	0.179***	18.92	0.190***	20.19
	(0.06)		(0.06)		(0.05)	
*PGDP*	0.330***	34.74	0.330***	34.88	0.342***	36.34
	(0.08)		(0.09)		(0.10)	
Urbanization	0.302***	31.79	0.298***	31.50	0.266***	28.27
	(0.08)		(0.09)		(0.09)	

Notes: Standard errors are presented in parentheses. The regression coefficient (β_*k*_) was calculated using equation (2), and the contribution was calculated using equation (3), as detailed in the Methods section. *, **, and *** represent significance levels of *p* < 0.10, 0.05, and 0.01, respectively.

From a regional perspective (Table 5), the influence of each indicator on the per capita years of education differed greatly among the regions, although *STR* had significant negative effects and the other indicators had positive effects in all regions. For the eastern and central regions, which have undergone more rapid economic development, the primary factor that affected education was economic growth (*PGDP*), with contributions of 34.0 and 37.0%, respectively. However, the relatively undeveloped western region has a low population density and a relatively underdeveloped transportation infrastructure, so urbanization has became the primary factor affecting education (a contribution of 52.6%). The expenditure on education in western China had less than half the effect it had in the eastern and central regions, and *STR* had a much lower effect in the western region than in the central region. This does not indicate that either factor is unimportant; rather, it reinforces the greater importance of urbanization.

**Table 5:** Regression results for the drivers of per capita years of education from a regional perspective over the period from 2000 to 2019. Regions are defined in Fig. 2. *STR*, student–teacher ratio; *PGDP*, per capita GDP.

	Eastern		Central		Western	
	Regression coefficient	Contribution (%)	Regression coefficient	Contribution (%)	Regression coefficient	Contribution (%)
*STR*	−0.124**	13.85	−0.211**	20.42	−0.165**	14.55
	(0.04)		(0.08)		(0.06)	
Expenditure	0.206*	23.02	0.248**	24.01	0.119*	10.49
	(0.09)		(0.08)		(0.06)	
*PGDP*	0.304***	33.97	0.382***	36.98	0.254	22.40
	(0.09)		(0.10)		(0.24)	
Urbanization	0.261***	29.16	0.192	18.59	0.596*	52.56
	(0.07)		(0.19)		(0.28)	

Notes: Standard errors are presented in parentheses. The regression coefficient (β_*k*_) was calculated using equation (2), and the contribution was calculated using equation (3), as detailed in the Methods section. *, **, and *** represent significance levels of *p* < 0.10, 0.05, and 0.01, respectively.

**Fig. 4:**
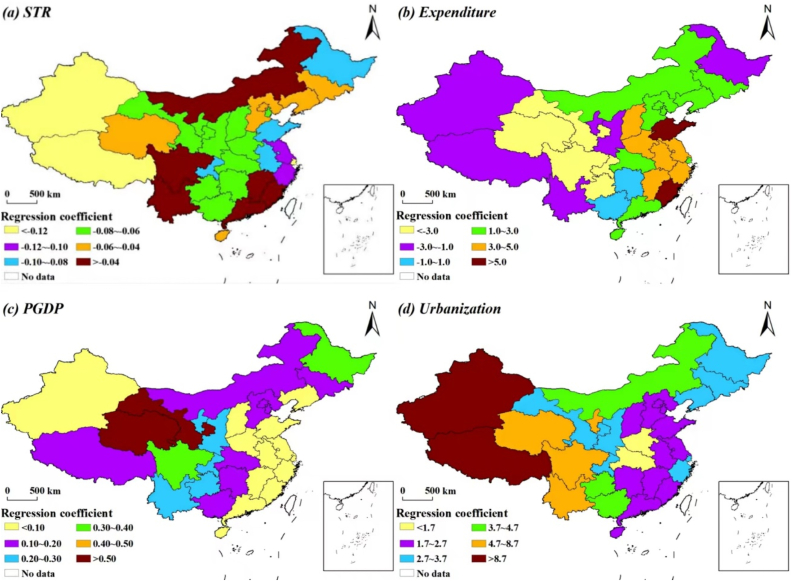
Distribution of the regression coefficients for the effects of the four explanatory variables based on the geographic and time-weighted regression (GTWR) model for each of the 31 Chinese provinces from 2000 to 2019. *PGDP*, per-capita GDP; *STR*, student–teacher ratio.

The results of the GTWR model (*R*^2^ = 0.94, *p* < 0.05) showed significant differences among the provinces in the degree of influence of the four explanatory factors on the per capita years of education (Fig. 4a–d). For example, *STR* had the strongest negative contribution in Xinjiang and Tibet, which indicates that the lack of teachers in the western region has had a strong negative effect on the per capita education level. The contributions of expenditure and *PGDP* differed widely among the provinces, and showed no clear pattern. In contrast, the influence of urbanization has generally decreased from west to east, which indicates that the population aggregation caused by urbanization had the greatest positive effect in Xinjiang and Tibet, which are sparsely populated.

## 5 Discussion

Education is one of the core elements that enhances a country's comprehensive national strength and cultural soft power, and is also concerned with improving the nation's standard of living and inter-generational sustainable development [29]. Our regression results clearly show that increasing the per capita investment in education and improving the economic level (*PGDP*) and urbanization have greatly promoted education, whereas an increasing *STR* can decrease educational attainment from both national and regional perspectives. Thus, when the goal is to improve education, we should not only invest our efforts in promoting economic development, increasing education funding, and promoting urbanization in an orderly manner, but should also pay attention to the training of teachers and to efforts to decrease the student–teacher ratio.

Among the factors influencing the per capita years of education, economic factors were most influential, and their contributions continued to increase over time (i.e., the β_*k*_ coefficients were significant and positive and showed a time-lag effect). This conclusion can be attributed in large part to the fact that rapid modernization has created a high demand for highly qualified workers [27], which motivates people to obtain more education so they can find better jobs and helps them compete with other workers [30]. Education is therefore an important factor in socioeconomic development, and because socioeconomic development promotes the development of education, the two processes are mutually supporting [31]. This suggests that governments should design their education policy based on the level of regional economic development, and should promote coordinated development and common progress of the two processes.

We found significant positive associations between education expenditure and the per capita years of education. However, despite China's increasing expenditure on education, a large gap remains between the per capita education expenditure and that of developed countries [32], and the problem of unequal and uneven regional distribution of education expenditures in China remains prominent [33]. The insufficient investment in education in less-developed regions exacerbates the risk that school-age youth in these regions will forego higher levels of education and hinders the cultivation of skills they will need in the workplace [26]. Increasing education funding in less-developed regions may bring higher marginal benefits to each additional year of education [8]. While increasing per capita spending on education, the government must also ensure the rationality, fairness, and effectiveness of its funding allocation.

The development of urbanization is also important for the progress of education. Urbanization shortens the distance between students and schools, reduces the cost of schooling, and, more importantly, greatly increases the accessibility of education in sparsely populated areas (e.g., western China) by increasing the concentration of people. The more developed and urbanized areas also have the economic resources to provide a better and well-funded education system [23], whereas less-developed and rural areas have a lower quality of education and lower student enrollment in their schools due to their disadvantaged position when competing with large cities for education funding and other resources [34]. The education gap between urban and rural residents will put rural residents in a disadvantaged position in the job market [35], which in turn increases the urban–rural income gap and may reduce investments in education, thereby exacerbating the gap between urban and rural residents and promoting feedback that creates a vicious circle [36]. To promote the development of education and decrease the gap between urban and rural areas, governments must increase their investment in education in rural areas while promoting urbanization in an orderly and sustainable manner, thereby facilitating student access to better educational facilities.

An inappropriately high student–teacher ratio significantly hinders the development of education. This situation not only increases the teacher's burden, but also dilutes the amount and quality of the attention they can give to each student, which inevitably decreases both the quality of education and student enthusiasm for learning [26]. High-quality education is more than simply encouraging students to attend school through political coercion and economic incentives; it requires a large number of trained teachers to educate and guide students [15]. Therefore, for countries and regions that are lagging behind developed countries and regions, it is still important to develop the economy and invest in education, but efforts to improve teacher salaries and increase the number of teachers to solve the problem of imbalanced *STR* should not be neglected.

Economic inputs, social development, and teacher resources have a continuous and pervasive effect on a student's willingness and ability to receive higher levels of education (e.g., based on their cognitive ability or financial ability). The time-lag effects we observed cause each of the factors that we studied to remain significantly related to a student's educational attainment in the long term; for example, the economic factors and educational inputs had an increasing degree of influence in the second and third years after they were implemented. When formulating policies to steadily promote sustainable development of education, countries and regions should be more concerned with sustained national economic development and investment in education than with short-term benefits. In addition, the magnitude of the effect of each factor on per capita educational attainment varies considerably among regions and provinces. The reason may lie in differences in a region's or province's natural resource endowment, geospatial structure, socioeconomic conditions, and degree of infrastructure development [12]. Such differences are common between both countries and regions, and are objective and persistent [37]. The existence of regional heterogeneity suggests that differentiated education policies tailored to the unique needs of each region, rather than monolithic "one size fits all" policies, are crucial for successful and sustainable development of education.

Education is more than an important component of students' skills and their quality of life; it is also a vital contributor to society and to socioeconomic development. At the same time, it serves as an important means of improving the student's quality of life and enhancing the skills that increase their ability to survive in a competitive job market, as well as their human rights [38]. Education is not only a major practical issue that must be addressed in China to achieve a long-term strategy for sustainable development [8,39], but also one of the core issues for every developing country that wants to improve living standards for their citizens and reduce inequality [27]. Improving educational conditions in countries around the world, especially in remote and backward regions, is an urgent need, and must attract the attention of both national governments and the international community. Different countries and regions should not only learn from our insights into the experience and lessons of China, but should also develop education policies tailored to their own local conditions that will improve the skills and quality of life of their citizens.

## 6 Conclusion and recommendations

By analyzing the factors that affect the educational attainment of Chinese nationals and the strength of each factor's influence, we identified and analyzed the key driving forces behind China's educational development and can make recommendations based on the results of our analyses. We found that the factor with the most influence on education was the economic development level, whose importance increased continuously over time, and that the education expenditure and urbanization level were also positively related to the per capita educational attainment, whereas the lack of teacher resources (a high student–teacher ratio) hinders the development of education. The strength of the effect of different factors varies considerably across regions, which suggests a need for region-specific solutions. Although our conclusions are not surprising, our novel method provides better support than previous research for the conclusions by examining a wider range of factors and a longer time period than previous research, while also providing a way to identify important differences between regions.

Based on the results of our study, we propose the following recommendations for the advancement of education. First, when formulating education policies in different regions, it is necessary to fully consider the local economic and social development conditions and drive the improvement of education conditions through economic development to form a mutually promoting relationship between education and economic development. Second, the relevant government departments should pay attention to the fairness of the regional distribution of educational resources while increasing the investment in education funds; they can accomplish this by increasing the support for less-developed regions. While promoting the level of urbanization in an orderly manner, attention must also be paid to education in rural areas to narrow the gap between urban and rural education. Third, the lack of teacher resources in less-developed countries and regions will seriously undermine the effectiveness of education, which indicates that we must attach more importance to the cultivation of high-quality teachers and allocate some of the best teachers to the backward regions through policy support and financial assistance. Finally, the existence of time-lag effects and regional heterogeneity in the strength of the factors affecting the level of education requires continuous attention when developing policy measures that are tailored to the unique demands of each region.

These conclusions provide theoretical support and practical references for China and other countries that are seeking to improve their delivery of education. Compared with previous studies, our research adds to the literature by providing a more in-depth analysis of the strengths of different factors that promote education and of the heterogeneity of these strengths among regions. However, due to a lack of consistent and comparable datasets across China, our analysis was limited to the provincial level and to variables for which comparable data existed in all provinces. When better data become available in the future, our analysis could be extended below the provincial level to county and city levels and could include more driving variables. In addition, researchers could extend our analysis to multiple countries to compare their key driving factors. We should also explore more sophisticated methods to clarify the mechanisms by which different factors work so as to improve the ability of our results to improve their support for policy development.

## Author contribution statement

S.C. conceived and designed the experiments and critically reviewed the manuscript’s intellectual content; L.Y. performed the experiments and wrote the main text of the manuscript after analyzing and interpreting the data. Both authors have reviewed the manuscript and approved it for submission.

## Additional information

No additional information is available for this paper.

## Declaration of competing interest

The authors declare no conflict of interest. The opinions expressed here are those of the authors and do not necessarily reflect the position of the Government of China or of any other organization. We consent to publish this article in your journal and to transfer its copyright to the publisher once the manuscript has been accepted.
